# Ethylene signaling via Ethylene Response Factors (ERFs) modifies wood development in hybrid aspen

**DOI:** 10.1186/1753-6561-5-S7-I15

**Published:** 2011-09-13

**Authors:** Judith Felten, Jorma Vahala, Jonathan Love, András Gorzsás, Lorenz Gerber, Manoj Kumar, Jaakko Kangasjärvi, Björn Sundberg

**Affiliations:** 1 Umeå Plant Science Center, Department of Forest Genetics and Plant Physiology, Swedish University of Agricultural Sciences, SE-901 83 Umeå, Sweden; 2 Department of Biosciences, Division of Plant Biology, University of Helsinki, PO Box 65, FI-00014 Helsinki, Finland

## Background

The phytohormone ethylene (ET) has the potential to regulate secondary growth of plants and wood formation in trees. Application of exogenous ethylene or its *in planta* precursor, 1-aminocyclopropane-1-carboxylic acid (ACC), to wood forming tissues of hybrid aspen (*Populus tremula x Populus tremuloides*) enhances xylem growth [1]. In the same study it was demonstrated that stimulation of enhanced xylem formation (tension wood, TW) at the upper side of leaning stems is mediated by endogenous ET. The production of endogenous ET in TW forming tissues is further supported by the increase of ACC oxidase gene transcript and enzyme activity on the TW side [2].

The ET perception and signal transmission cascade in Arabidopsis has been linked to the transcriptional activation of Ethylene Response Factors (ERFs) [3,4]. As transcription factors, ERFs regulate the expression of various specific downstream target genes by binding to *cis*-elements in their promoters [5]. We hypothesize that ERFs participate in xylem development through ethylene signaling and that they are involved in ET responses during TW formation.

## Results and conclusions

We identified 169 *ERF* genes in the *Populus trichocarpa* genome versions 2.0 and 2.2 using regular expression method and pfam search for the ERF domain. These ERFs grouped into 11 distinct groups, similar to ERFs identified in Arabidopsis and rice [6]. Using qPCR we showed that a majority of the *ERF* transcripts were detectable in stem tissues of in vitro or greenhouse grown hybrid aspen. The responsiveness of all *ERF*s to short term ACC treatments (10h, in vitro plants) and to short or long term ethylene treatments (24 or 2 weeks, greenhouse grown plants), was assessed by qPCR. Most of the *ERF*s responded to at least one of the treatments, mostly by increased transcript accumulation. We identified *ERF*s that were specifically induced within the early ACC/ET- or the late ET-response. In addition, some *ERF*s showed prolonged induction up to 2 weeks of ET treatment. These different transcript patterns indicate that different *ERF*s may be involved in distinct, temporally distinguished processes during the ACC/ET- induced secondary xylem growth response.

According to their expression and capacity to be induced by ET or ACC, we selected 26 *ERF* candidates and investigated whether those were responsive to endogenous ET-signals in leaned stems during TW formation. We compared the induction of those 26 candidate *ERF*s in TW with *ERF* accumulation during long-term (2 weeks) ET treatment. Interestingly, a significant overlap of *ERF* induction in both conditions was found. From the 20 *ERF*s that were induced after long-term ET treatment, 16 had an increased transcript abundance during tension wood formation. This indicates that on an *ERF* transcript basis, tension wood formation is largely comparable to a long-term ET treatment.

Based on the transcript data, twenty ERFs were selected for overexpression in hybrid aspen cambium/xylem under the *pLMX5* promoter [1]. Successful overexpression of the selected *ERF*s in transgenic plants was confirmed and two to seven lines of each overexpressed ERF were phenotyped in a greenhouse trial. In general, *ERF* overexpression caused only mild alterations of overall plant stature (height and radial growth). Only overexpression of one *ERF* candidate led to a severe dwarf phenotype with thin stems, reduced fiber and vessel size, reduced height growth and smaller leaves. The absence of any striking phenotypes in all other plants suggests that other regulators in addition to the overexpressed *ERF*s may be necessary to mimic the enhanced growth response observed during ET/ACC/TW mediated stimulation of secondary xylem growth.

A Fourier-Transformed Infrared spectroscopic and Pyrolysis GC-MS based screening of five lines for each of the 20 overexpressed ERFs revealed that five *ERF*s led to changes in cell wall composition in xylem tissues when overexpressed. This suggests that these ERFs have the ability to modify cell wall composition in wood forming tissues and may regulate the expression of cell-wall biosynthesis genes. This hypothesis is now under further investigation.
